# Effect of Schistosomiasis and Soil-Transmitted Helminth Infections on Physical Fitness of School Children in Côte d'Ivoire

**DOI:** 10.1371/journal.pntd.0001239

**Published:** 2011-07-19

**Authors:** Ivan Müller, Jean T. Coulibaly, Thomas Fürst, Stefanie Knopp, Jan Hattendorf, Stefanie J. Krauth, Katarina Stete, Aurélie A. Righetti, Dominik Glinz, Adrien K. Yao, Uwe Pühse, Eliézer K. N'Goran, Jürg Utzinger

**Affiliations:** 1 Department of Epidemiology and Public Health, Swiss Tropical and Public Health Institute, Basel, Switzerland; 2 Department of Public Health, Institute for Sports and Sports Sciences, University of Basel, Basel, Switzerland; 3 Centre Suisse de Recherches Scientifiques en Côte d'Ivoire, Abidjan, Côte d'Ivoire; 4 Université de Cocody, Abidjan, Côte d'Ivoire; 5 University of Basel, Basel, Switzerland; 6 Faculty of Medicine, Albert-Ludwigs-University of Freiburg, Freiburg im Breisgau, Germany; 7 Human Nutrition Laboratory, Institute of Food, Nutrition, and Health, Swiss Federal Institute of Technology Zurich, Zurich, Switzerland; 8 Services de Santé Scolaire et Universitaire, Agboville, Côte d'Ivoire; Ministry of Health, Uganda

## Abstract

**Background:**

Schistosomiasis and soil-transmitted helminthiasis are important public health problems in sub-Saharan Africa causing malnutrition, anemia, and retardation of physical and cognitive development. However, the effect of these diseases on physical fitness remains to be determined.

**Methodology:**

We investigated the relationship between schistosomiasis, soil-transmitted helminthiasis and physical performance of children, controlling for potential confounding of *Plasmodium* spp. infections and environmental parameters (i.e., ambient air temperature and humidity). A cross-sectional survey was carried out among 156 school children aged 7–15 years from Côte d'Ivoire. Each child had two stool and two urine samples examined for helminth eggs by microscopy. Additionally, children underwent a clinical examination, were tested for *Plasmodium* spp. infection with a rapid diagnostic test, and performed a maximal multistage 20 m shuttle run test to assess their maximal oxygen uptake (VO_2_ max) as a proxy for physical fitness.

**Principal Findings:**

The prevalence of *Schistosoma haematobium*, *Plasmodium* spp., *Schistosoma mansoni*, hookworm and *Ascaris lumbricoides* infections was 85.3%, 71.2%, 53.8%, 13.5% and 1.3%, respectively. Children with single, dual, triple, quadruple and quintuple species infections showed VO_2_ max of 52.7, 53.1, 52.2, 52.6 and 55.6 ml kg^−1^ min^−1^, respectively. The VO_2_ max of children with no parasite infections was 53.5 ml kg^−1^ min^−1^. No statistically significant difference was detected between any groups. Multivariable analysis revealed that VO_2_ max was influenced by sex (reference: female, coef. = 4.02, p<0.001) and age (years, coef. = −1.23, p<0.001), but not by helminth infection and intensity, *Plasmodium* spp. infection, and environmental parameters.

**Conclusion/Significance:**

School-aged children in Côte d'Ivoire showed good physical fitness, irrespective of their helminth infection status. Future studies on children's physical fitness in settings where helminthiasis and malaria co-exist should include pre- and post-intervention evaluations and the measurement of hemoglobin and hematocrit levels and nutritional parameters as potential co-factors to determine whether interventions further improve upon fitness.

## Author Summary

The burden of parasitic worm infections is considerable, particularly in developing countries. It is acknowledged that parasitic worm infections negatively impact on children's school performance and physical development. A deeper understanding of these linkages is important for updating burden of disease measures. We investigated the relationship between worm infection status and physical fitness of 156 school children from Côte d'Ivoire and controlled for potential confounding of *Plasmodium* infection (the causative agent of malaria) and environmental parameters (temperature and humidity). Children were diagnosed for parasitic worm and *Plasmodium* infections, examined by a physician, and participated in a 20 m shuttle run test to assess their maximal oxygen uptake (VO_2_ max) as a proxy for physical fitness. Most of the children had parasitic worms and a *Plasmodium* infection. Nevertheless, their physical fitness was excellent (average VO_2_ max: 52.7 ml kg^−1^ min^−1^). The level of VO_2_ max was only influenced by sex and age, but not by parasitic worms and *Plasmodium* infections. In future studies, the dynamics of children's physical performance should be assessed before and after control interventions, including the assessment of blood hemoglobin, hematocrit, and nutritional indicators to determine whether physical fitness in worm- and *Plasmodium*-infected individuals can be further improved.

## Introduction

Neglected tropical diseases and malaria are widespread on the African continent and elsewhere in the developing world. These diseases predominantly plague the poorest of the poor and delay their social and economic development [1]–[3]. Infections with soil-transmitted helminths (*Ascaris lumbricoides*, hookworm, and *Trichuris trichiura*) and schistosomes (*Schistosoma mansoni* and *S. haematobium*), for example, affect hundreds of millions of people with untold morbid sequelae [4]–[6]. Helminths parasitizing humans can destroy the organs and tissues in which they live and compete for nutrients with the human host. Consequently, infections can result in abdominal pain, diarrhea, intestinal obstruction, anemia, malnutrition, ulcers and, particularly in severe chronic and untreated infections, even death [5], [7]–[9]. These sequelae, in a chronic stage, may retard children's physical development [8], [10], [11]. It is also hypothesized that helminth infections negatively impact on cognitive abilities, and hence on children's school performance [12]. However, some more recent studies could not find any clear association between helminth infections and school performance, and hence additional research is needed [13]–[15].

To date, only few attempts have been made to quantify the effect of schistosomiasis and soil-transmitted helminth infections on children's physical fitness [16], [17]. In the early stage of a schistosome infection, general fatigue is a common symptom described in various age groups [18]. As helminth infections progress, the intensity and duration is thought to play an important role in influencing physical fitness [19]. Hence, the assessment of infection intensities should be regarded as an important component of evaluating the effect of helmintic diseases on people's general health and wellbeing. In the light of the currently ongoing comprehensive revision of the global burden of diseases estimates [20], [21] and the crucial role of disability weights, which are exceedingly difficult to estimate among the neglected tropical diseases [22]–[24], the quantitative impact of helminth infections on humans' physical fitness deserves closer attention.

The aim of this study was to investigate whether or not there is a relationship between helminth infection status among school-aged children and their physical fitness. The study was carried out in Côte d'Ivoire, in an area highly endemic for schistosomiasis and, to a lesser extent, soil-transmitted helminthiasis, using a cross-sectional study type. Malaria is co-endemic, and hence we also determined *Plasmodium* spp. infections and controlled for environmental factors such as ambient air temperature and humidity.

## Methods

### Ethics statement

The study protocol was approved by the institutional research commission of the Swiss Tropical and Public Health Institute (Basel, Switzerland) and received clearance from the “Ethikkommission beider Basel” (EKBB, reference no. 377/09) and the Comité National d'Ethique et de la Recherche (CNER) in Côte d'Ivoire (no. 1993 MSHP/CNER). The study was covered by an insurance company (GNA Assurance; Abidjan, Côte d'Ivoire, policy no. 30105811010001). District health and education authorities, the village chief, parents/guardians, and school children were informed about the purpose, procedures, and potential risks and benefits of the study. Written informed consent was obtained from parents/guardians and children assented orally. Participation was voluntary and children could withdraw from the study anytime without further obligation. All results were coded and treated confidential. In some cases, where a need for medical intervention was required, the name of the individual was communicated to the local health service in order to provide appropriate medical follow-up. At the end of the study, all children attending the primary school of *Grand Moutcho I, II* and *III* were administered praziquantel (single 40 mg/kg oral dose) and albendazole (single 400 mg oral dose) free of charge, irrespective of the children's helminth infection status.

### Study design and sample size calculation

We conducted a cross-sectional survey on physical activity in school children. We assumed that the arithmetic mean of the maximal oxygen uptake (VO_2_ max), which was measured as a proxy for the school-aged children's physical fitness, would be 50 ml kg^−1^ min^−1^ with a standard deviation of 5 ml kg^−1^ min^−1^ (see σ in formula 1) [25]. Moreover, we assumed that a difference of VO_2_ max of 5% (i.e., 2.5 ml kg^−1^ min^−1^; see *D* in formula 1) is of clinical relevance and that the ratio of children with the predominant helminth species *versus* non-infected children would be roughly 1∶1. According to formula (1) given by Eng (2003) [26]

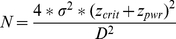
(1)and allowing for 10% non-compliance, we calculated that 186 children in total would need to be enrolled to achieve a power of 90% at an alpha error of 5% to find a statistical significance in VO_2_ max between helminth-infected and non-infected individuals. Finally, we aimed to enroll 200 children to account for imbalances in the study group sizes.

### Study area and population

The study was carried out between January and April 2010 in the primary school *Grand Moutcho*, located in the health district of Agboville, south Côte d'Ivoire (geographical coordinates: 05°56′0″ N latitude, 04°13′0″ W longitude). The study area is located 76 km north of Abidjan, the economic capital of Côte d'Ivoire, at an altitude of below 100 m above sea level. In south Côte d'Ivoire, the vegetation primarily consists of rainforest, the relief is flat and the long and short rainy seasons occur between April and July and from mid-September to November, respectively. During the school year 2009/2010, there were a total of 204 children attending grades 4, 5, and 6 in *Grand Moutcho II* and *III*, and hence all of them were invited to participate in the study.

### Field and laboratory procedures

Village authorities and teachers were informed about the purpose and procedures of the study. Subsequently, teachers were asked to prepare class lists with the name, age, and sex of the children attending grades 4–6 of *Grand Moutcho II* and *III*. After written informed consent was obtained from parents or guardians and children gave oral assent to participate, children were provided with plastic containers labeled with unique identification numbers and invited to submit a small portion of their own fresh morning stool the following day. Stool samples were collected between 08:00 and 10:00 hours and children were handed out a new empty container for urine collection starting at 10:00 hours. This procedure was repeated the following day in order to obtain two stool and two urine samples from each child.

Stool and urine samples were transferred to a nearby laboratory in the district town Agboville. For the diagnosis of *S. mansoni* and soil-transmitted helminth infections, duplicate Kato-Katz thick smears, using 41.7 mg templates [27], were prepared from each stool sample and examined under a microscope by experienced laboratory technicians. The number of eggs was counted and recorded for each helminth species separately. For the diagnosis of *S. haematobium*, urine samples were subjected to the filtration method [28], [29]. In brief, 10 ml of vigorously shaken urine were pressed through a small-meshed filter (30 µm), a drop of lugol solution was added to the filter paper on the microscope slide, and the slides were examined quantitatively under a microscope for *S. haematobium* eggs by experienced technicians. Consistent with our previous work, 10% of the slides were re-examined by a senior technician [30], [31] and, in case of disagreement, the results were discussed with the concerned technician and the corresponding samples read a third time and used as a reference.

### Physical examination and shuttle-run test

Children who had provided two stool and two urine samples were clinically examined by a physician three days after the laboratory examination and, based on observed signs and symptoms, checked for their general state of health. Additionally, a rapid diagnostic test (RDT) for malaria was performed (ICT ML01 malaria Pf kit; ICT Diagnostics, Cape Town, South Africa). Children with clinical malaria (defined as positive RDT plus recent history of fever), asthma (assessed by study physician using a stethoscope), anemia (assessed by study physician after pulling down of eyelid and noting pale color [32]), or dyspnea (assessed by study physician using a stethoscope), according to the physician's appraisal, were excluded from the subsequent fitness test as motivating them to reach their maximal physical capacity was considered as potentially harmful.

The aim of the physical fitness test was to measure children's aerobe capacity and maximal oxygen up-take, the so-called VO_2_ max [33]. The maximal multistage 20 m shuttle run test [34]–[36] is considered to be reliable and valid and was therefore utilized to determine the maximal aerobic capacity of the school children. Seventeen groups with a maximum of 10 children per group were running one group after the other on three consecutive days between 08:30 and 11:30 hours and between 16:00 and 18:00 hours. While doing the 20 m shuttle run test, the maximal heart rate of participating children was assessed using a Polar RS400 watch (Polar Electro Europe BV; Zug, Switzerland) to ensure that children really tried to reach their maximal physical capacity. Achieving less than 180 heart beats per min was taken as criterion that a child did not perform the test until the physical capacity limit.

To guarantee the comparability of the physical tests and to minimize external influences, which might affect the different test series of the 20 m shuttle run, ambient air temperature and humidity were monitored with a thermometer and a hygrometer, respectively.

### Statistical analysis

Parasitological data were double-entered and cross-checked in Access version 2007 (Microsoft Corp.; Redmond, WA, USA) and analyzed in STATA version 10.1 (STATA Corp.; College Station, TX, USA). The completed race distance (levels and shuttles) according to the 20 m shuttle run test were obtained from version 3.2 of the Team Beep Test 20 m software (RobJWood Designs; Mount Hawthorn, Australia).

For each child, the arithmetic mean of the helminth species-specific egg counts from the four Kato-Katz thick smears was calculated and multiplied by a factor 24 to obtain a standardized measure of infection intensity, expressed as eggs per gram of stool (EPG). Helminth infection intensities were classified into light, moderate, and heavy, according to World Health Organization (WHO) guidelines [29], [37]. The upper limits of light and moderate infections were 100 and 400 EPG for *S. mansoni*; 2,000 and 4,000 EPG for hookworm; and 5,000 and 50,000 EPG for *A. lumbricoides*, respectively. *S. haematobium* egg counts were classified into light (<50 eggs/10 ml of urine) and heavy (≥50 eggs/10 ml of urine or visible hematuria) [37].

Physical fitness data were gathered and analyzed according to standard methodologies put forth in “The Guidelines for Exercise Testing and Prescription” from the American College of Sports Medicine (ACSM) [33]. VO_2_ max results were obtained by using the age-adjusted (*X_1_* = age in years) positive linear relation between the shuttle running speed (*X_2_* = speed in km/h) and VO_2_ max as expressed by Léger & Mercier [36] in equation 2.
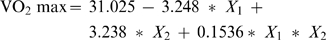
(2)


Only those children who had complete data records (i.e., written informed consent, four Kato-Katz thick smears, two urine filtrations, a RDT for malaria, completed clinical examination and 20 m shuttle run test) were included in the final analysis. Arithmetic mean, χ^2^ and *t*-test statistics, as well as univariate and multivariable regression analyses were employed to assess statistical significance (p<0.05). Children with complete parasitological and clinical data, but no valid results from the physical fitness test due to exclusion in the clinical examination or an invalid maximum heart rate while completing the physical fitness test were included in an attrition analysis.

## Results

### Compliance and demographic results

**Figure 1:**
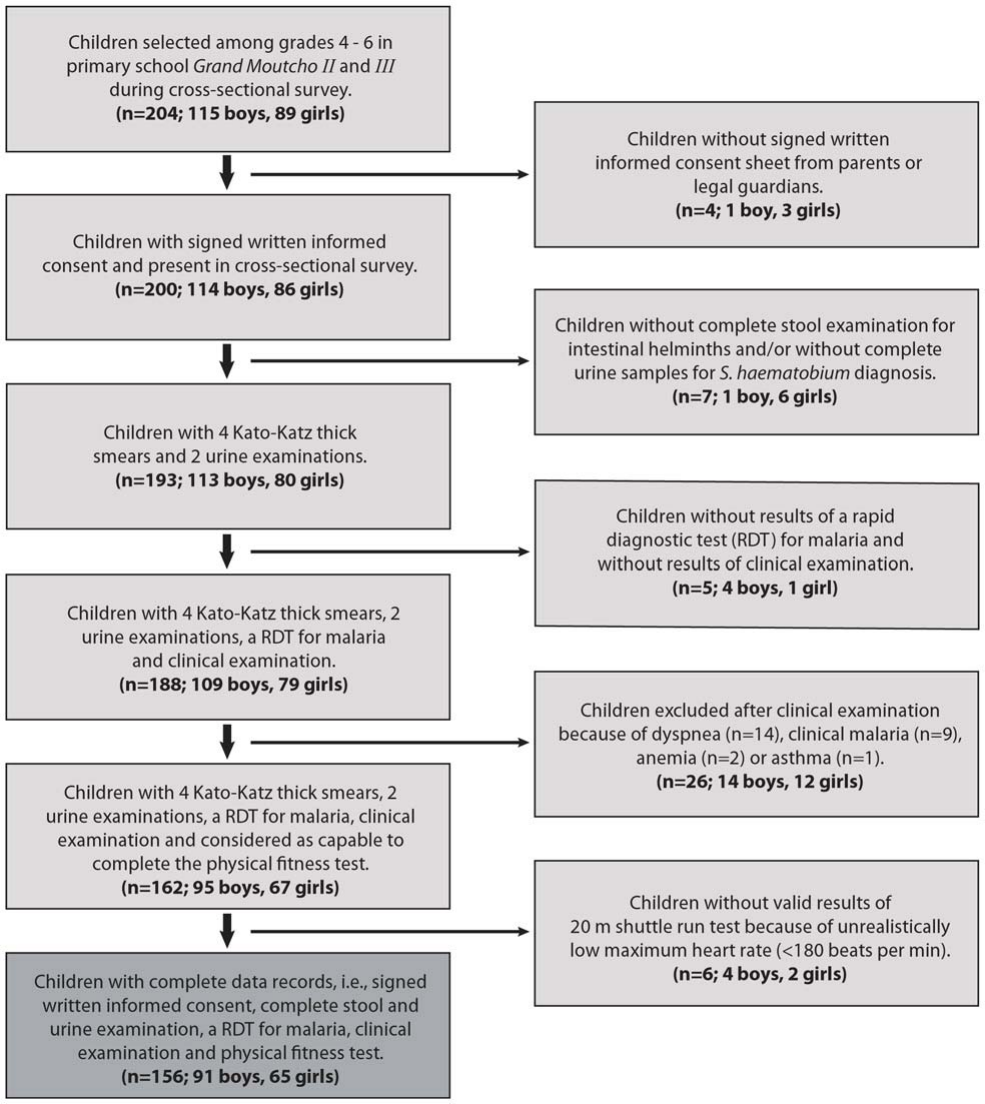
Study participation and compliance. Diagram showing the study participation and compliance of school children attending grades 4-6 of the primary school *Grand Moutcho II* and *III*, near Agboville, a rural community of south Côte d'Ivoire in early 2010.

All 204 school children attending grades 4–6 of *Grand Moutcho II* and *III* were invited to participate in the study. As shown in Figure 1, 200 children (98.0%) returned written informed consent sheets signed by their parents/guardians. Complete parasitological results (i.e., four Kato-Katz thick smears, two urine filtrations, and one RDT for malaria) were available from 188 children and they all took part in the clinical examination and were willing to perform the 20 m shuttle run test. Hence, the compliance rate was 92.2%. However, another 26 children were excluded from the 20 m shuttle run test according to the physician's judgment. Exclusion criteria were dyspnea (n = 14), clinical malaria (n = 9), anemia (n = 2), and asthma (n = 1). The remaining 162 children participated in the 20 m shuttle run test, but the results of six children were considered as invalid because of maximum heart rate below 180 heart beats per min. Hence, the final study cohort consisted of 156 children (76.5% of the initial 204).

The median age of the final study population was 12 years with a range of 7–15 years. However, most of the children were aged between 9 and 15 years (98.7%). The predominant age-group were 13-year-old (n = 39). There were more boys (n = 91) than girls (n = 65).

### Physical fitness

The overall arithmetic mean of the VO_2_ max values of the 156 children was 52.7 ml kg^–1^ min^−1^ (95% confidence interval (CI): 52.0–53.4 ml kg^−1^ min^−1^) (Table 1). Extreme values (minimum and maximum) were at 40.5 and 60.6 ml kg^−1^ min^−1^. While girls had a mean VO_2_ max of 50.4 ml kg^−1^ min^−1^ (95% CI: 49.4–51.3 ml kg^–1^ min^−1^), the respective value for boys was significantly higher at 54.4 ml kg^−1^ min^−1^ (95% CI: 53.5–55.2 ml kg^−1^ min^−1^). The observed differences in mean VO_2_ max values between girls and boys varied with age. In general, mean VO_2_ max values gradually decreased with age; for boys it decreased from 56.8 ml kg^−1^ min^−1^ among 7-year-old to 48.8 ml kg^−1^ min^−1^ among those aged 15 years. The respective decrease in girls was from 54.2 to 44.7 ml kg^−1^ min^−1^.

**Table 1:** Comparison of age- and sex-specific mean VO_2_ max values among Ivoirian and Canadian children.

Age	Sex	Agboville, Côte d'Ivoire, 2010			Québec, Canada, 1981		
(years)		n	Mean VO_2_ max	95% CI	n	Mean VO_2_ max	95% CI^a^
7	M	2	56.8	15.7-97.9	297	51.2	50.9-51.6
	F	0	n.a.	n.a.	299	50.3	50.0-50.6
8	M	0	n.a.	n.a.	303	51.7	51.2-52.1
	F	0	n.a.	n.a.	308	49.8	49.4-50.2
9	M	5	55.9	53.3-58.4^b^	322	51.5	51.1-52.0^b^
	F	6	54.2	52.2-56.1^b^	322	49.2	48.9-49.6^b^
10	M	12	56.0	53.3-58.8^b^	404	51.6	51.2-52.1^b^
	F	14	53.8	52.0-55.6^b^	335	46.8	46.5-47.1^b^
11	M	9	56.3	53.9-58.8^b^	386	51.1	50.7-51.6^b^
	F	7	50.2	47.1-53.3	382	47.5	47.1-47.9
12	M	24	54.5	53.0-56.0^b^	341	51.9	51.4-52.5^b^
	F	9	50.2	47.9-52.5^b^	292	46.7	46.2-47.1^b^
13	M	22	54.4	52.8-55.9^b^	325	50.1	49.5-50.7^b^
	F	17	48.4	47.0-49.8^b^	298	44.4	43.9-45.0^b^
14	M	10	52.9	50.5-55.3	289	50.1	49.5-50.7
	F	8	48.9	45.7-52.1^b^	260	41.7	41.1-42.2^b^
15	M	7	48.8	42.9-54.7	333	50.2	49.6-50.9
	F	4	44.7	39.0-50.3	260	41.2	40.5-41.8
7-15	M	91	54.4	53.5-55.2	3000	51.1	n.a.
7-15	F	65	50.4	49.4-51.3	2756	46.6	n.a.
7-15	Both	156	52.7	52.0-53.4	5756	48.9	n.a.

VO_2_ max values (expressed in ml kg^-1^ min^-1^) were obtained from 20 m shuttle run tests performed by 156 children attending grades 4-6 in the primary school of *Grand Moutcho II* and *III* near Agboville, south Côte d'Ivoire in early 2010 (present study) and from children in Québec, Canada, in 1981.

CI, confidence interval; F, female; M, male; n.a., not applicable.

a Values calculated by authors of the present article, based on data in Léger et al. (1988) [25].

b Statistically significant difference between the two studies according to non-overlapping 95% CI.

### Parasitological characteristics in relation to VO_2_ max

Prevalence and infection intensity of helminth and *Plasmodium* spp. infections, stratified by age and sex, are summarized in Table S1. Overall prevalences for *S. haematobium*, *Plasmodium* spp., *S. mansoni*, hookworm and *A. lumbricoides* were 85.3%, 71.2%, 53.8%, 13.5% and 1.3%, respectively. No eggs of *T. trichiura* were identified, whereas eggs of *Hymenolepis diminuta* were found in the stool of one child.

Among the 133 children infected with *S. haematobium*, 57.9% carried light and 42.1% heavy infections. The arithmetic mean egg count was 52 eggs/10 ml of urine (range: 1–346 eggs/10 ml of urine). Among the 84 *S. mansoni*-infected children, 64.3% presented with light, 32.1% with moderate, and 3.6% with heavy infection intensity. The arithmetic mean fecal egg count (FEC) of all *S. mansoni*-infected children was 116 EPG (range: 6–852 EPG). All 21 hookworm infections were diagnosed as light, with an arithmetic mean FEC of 42 EPG (range: 6–120 EPG). Only one light (4668 EPG) and one moderate (11,226 EPG) *A. lumbricoides* infection was detected.

VO_2_ max values of children with a *S. haematobium*, *S. mansoni*, hookworm or *A. lumbricoides* infection were 52.5, 52.2, 54.8 or 52.3 ml kg^−1^ min^−1^, respectively, whereas children without helminth infection showed a mean VO_2_ max of 52.9 ml kg^−1^ min^−1^ (Table 2). Multi-parasitism was very common. While only 3.8% of all children were neither infected with helminths nor with *Plasmodium* spp., 16.7% harbored one, 35.9% two, 38.5% three, 4.5% four and 0.6% even five parasite species concurrently. Children with single, dual, triple, quadruple and quintuple species infections showed VO_2_ max values of 52.7, 53.1, 52.2, 52.6 and 55.6 ml kg^−1^ min^−1^, respectively. The exact parasite combinations and respective VO_2_ max values are presented in Table 3.

**Table 2:** Helminth infection intensities in accordance with WHO guidelines [37] and mean VO_2_ max values.

Infection	Intensity	n	VO_2_ max	
			Mean	95% CI
No helminth infection	---	17	52.9	51.1-54.7
*S. haematobium*	All	133	52.5	51.8-53.4
	Light (<50 eggs/10 ml of urine)	77	52.8	51.8-53.8
	Heavy (≥50 eggs/10 ml of urine or visible hematuria)	56	52.2	50.9-53.5
*S. mansoni*	All	84	52.2	51.1-53.3
	Light (1-99 EPG)	54	52.8	51.4-54.2
	Moderate (100-399 EPG)	27	51.2	49.3-53.1
	Heavy (≥400 EPG)	3	51.4	37.0-65.8
Hookworm	All	21	54.8	52.8-56.8
	Light (1-1999 EPG)	21	54.8	52.8-56.8
	Moderate (2000-3999 EPG)	0	n.a.	n.a.
	Heavy (≥4000 EPG)	0	n.a.	n.a.
*A. lumbricoides*	All	2	52.3	10.4-94.2
	Light (1-4999 EPG)	1	55.6	n.a.
	Moderate (5000-49,999 EPG)	1	49.0	n.a.
	Heavy (≥50,000 EPG)	0	n.a.	n.a.

VO_2_ max values (expressed in ml kg^-1^ min^-1^) were achieved from 20 m shuttle run tests performed by 156 children attending grades 4-6 in the primary school of *Grand Moutcho II* and *III* near Agboville, a rural community of south Côte d'Ivoire in early 2010.

CI, confidence interval; EPG, eggs per gram of stool; n.a., not applicable.

**Table 3:** Multiparasitism and associated mean VO_2_ max values.

Infection	Parasites	No. of observations (%)	VO_2_ max	
			Mean	95% CI
No parasite infection	---	6 (3.8)	53.5	49.6-57.4
Single infection	All single infections	26 (16.7)	52.7	51.4-54.0
	*S. haematobium*	13 (8.3)	53.1	51.5-54.7
	*S. mansoni*	2 (1.3)	50.9	8.2-93.5
	*Plasmodium* spp.	11 (7.1)	52.5	50.2-54.9
Double infection	All double infections	56 (35.9)	53.1	51.9-54.3
	*S. haematobium* and *S. mansoni*	17 (10.9)	53.1	50.6-55.6
	*S. haematobium* and hookworm	2 (1.3)	51.7	36.3-67.1
	*S. haematobium* and *Plasmodium* spp.	34 (21.8)	52.8	51.4-54.3
	*S. mansoni* and hookworm	1 (0.6)	60.6	n.a.
	*S. mansoni* and *Plasmodium* spp.	1 (0.6)	52.6	n.a.
	Hookworm and *Plasmodium* spp.	1 (0.6)	58.2	n.a.
Triple infections	All triple infections	60 (38.5)	52.2	50.8-53.5
	*S. haematobium*, *S. mansoni* and hookworm	3 (1.9)	54.2	36.2-72.2
	*S. haematobium*, *S. mansoni* and *A. lumbricoides*	1 (0.6)	49.0	n.a.
	*S. haematobium*, *S. mansoni* and *Plasmodium* spp.	50 (32.1)	51.5	50.1-53.0
	*S. haematobium*, hookworm and *Plasmodium* spp.	5 (3.2)	57.1	53.0-61.5
	*S. mansoni*, hookworm and *Plasmodium* spp.	1 (0.6)	56.9	n.a.
Quadruple infections	All quadruple infections	7 (4.5)	52.6	48.4-56.9
	*S. haematobium, S. mansoni,* hookworm			
	and *Plasmodium* spp.	7 (4.5)	52.6	48.4-56.9
Quintuple infections	All quintuple infections	1 (0.6)	55.6	n.a.
	*S. haematobium, S. mansoni,* hookworm,			
	*A. lumbricoides* and *Plasmodium* spp.	1 (0.6)	55.6	n.a.

VO_2_ max values were obtained from 20 m shuttle run tests performed by 156 school children from the primary school in *Grand Moutcho*, Agboville, Côte d'Ivoire, in early 2010.

CI, confidence interval; n.a., not applicable.

**Figure 2:**
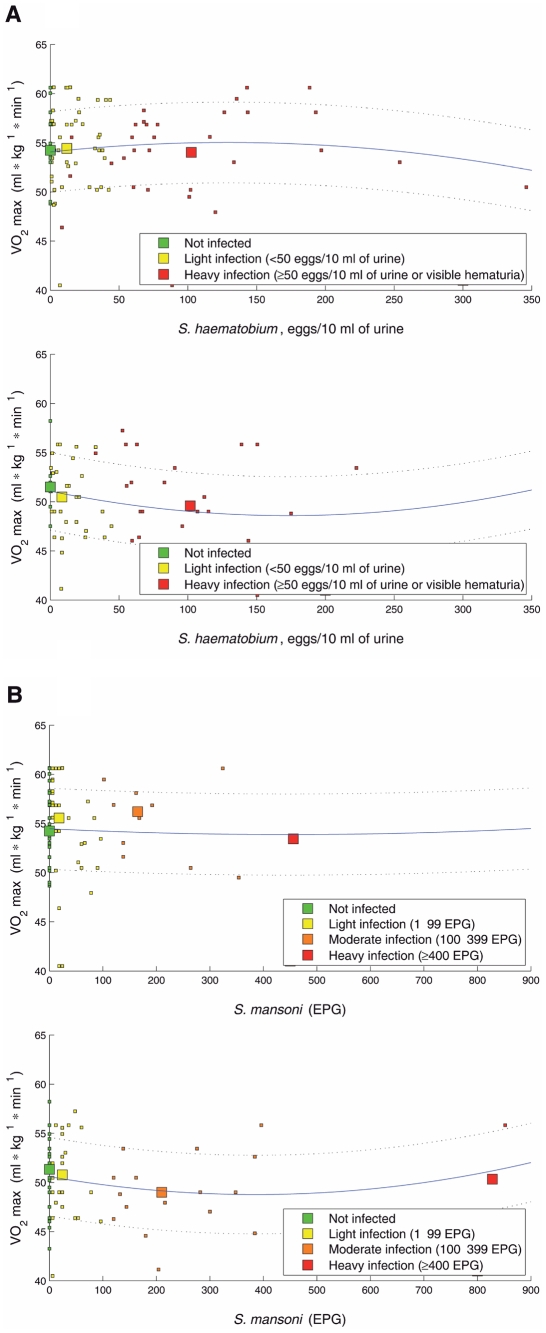
Sex-specific scatter plots of VO_2_ max values among Ivoirian school children. VO_2_ max values were obtained from 156 children attending grades 4-6 in the primary school of *Grand Moutcho II* and *III* near Agboville, south Côte d'Ivoire in early 2010 after performing a 20 m shuttle run test. Data are shown in accordance with children's infection status of *S. haematobium*, measured in number of eggs per 10 ml of urine (A), and *S. mansoni*, measured in number of eggs per gram of stool (EPG) (B). Scatter plots on the top represent males and scatter plots on the bottom females. Second order polynomial regression lines (solid lines) and their 95% confidence intervals (dotted lines) are presented.

However, as demonstrated by overlapping 95% CIs in Tables 2 and 3, no significant differences were found in the VO_2_ max values of helminth-infected and non-infected children, regardless of the helminth species investigated, regardless of whether children were infected with one or multiple species concurrently, and regardless of the helminth infection intensity. The results of the two parasites with the most diverse infection intensities, as measured by the number of eggs in a given amount of urine or stool, namely *S. haematobium* and *S. mansoni*, were used to illustrate their effect on the children's VO_2_ max. As documented in Figure 2, no clear trend was observable.

### Multivariable regression analyses with physical fitness as outcome

A multivariable regression analysis supported our findings from the descriptive statistics. After adjusting for temperature (range: 31–43°C) and relative humidity (range: 42–69%) of the ambient air (measures taken when the children performed the physical activity test), sex and age, differences in VO_2_ max values between children with differing parasitic infection status were not statistically significant (Table 4). The only statistically significant explanatories remained sex (reference: female, coeff. = 4.02, p<0.001) and age (coeff. = −1.23, p<0.001). These findings were robust and whether we used helminth infection intensity categories as defined by WHO or exact FECs had no influence on the outcome of the multivariable regression model.

**Table 4:** Multivariable regression analysis between VO_2_ max values and air temperature, humidity, sex, age, and infection status as explanatory variables.

		Multivariable regression analysis^a^		
Explanatory variables		Coef.	95% CI	P-value
Air temperature (in °C)		0.33	-0.16–0.83	0.185
Relative humidity of the air (in %)		0.09	-0.13–0.31	0.404
Sex (reference: female)		4.02	2.83–5.21	<0.001
Age (in years)		-1.23	-1.56–-0.89	<0.001
Malaria (reference: not infected)		-0.02	-1.31–1.26	0.973
Helminth infection (reference: not infected)				
*S. haematobium*	Light	-2.18	-10.37–6.00	0.599
	Heavy	-2.11	-10.45–6.23	0.618
*S. mansoni*	Light	-1.57	-9.52–6.38	0.697
	Moderate	-2.49	-10.34–5.36	0.532
	Heavy	-2.59	-11.35–6.16	0.559
Hookworm	Light	1.14	-6.36–8.64	0.764
No. of concurrent helminth infections (reference: 0)				
One		3.95	-4.41–12.30	0.352
Two		5.33	-10.46–21.12	0.506
Three		4.90	-17.63–27.42	0.668

VO_2_ max (ml kg^−1^ min^−1^) values resulting from 20 m shuttle run tests performed by 156 children attending grades 4–6 in the primary school of *Grand Moutcho II* and *III* near Agboville, a rural community of south Côte d'Ivoire in early 2010. Only explanatories with n>1 observations were included.

a Key indicators of the multivariable regression model: F (14, 140) = 8.20, p<0.001; R-squared = 0.450.

### Attrition analysis

Characteristics of the 32 children with complete parasitological and clinical data, but no results from the physical fitness test were compared with the 156 children comprising the final study sample. This attrition analysis revealed that the two groups were similar with regard to the proportions of girls (43.8% *vs*. 41.7%) and mean age (11.7 *vs*. 12.0 years). No statistically significant differences in helminth infection intensity categories were detected for *S. haematobium*, *S. mansoni*, hookworm and *A. lumbricoides* (all p>0.05), and occurrence of multiple helminth infections was comparable (p = 0.928) in both groups.

## Discussion

Only few attempts have been made to determine the effect of schistosomiasis and soil-transmitted helminth infections on children's physical performance, which is closely related to their general health and wellbeing, and hence a proxy measure of disability and disease burden. We investigated the relationship between helminth infection status and physical fitness in school children from Côte d'Ivoire and controlled for potential confounding of malaria and environmental influences. *Schistosoma* spp. and *Plasmodium* spp. infection were present in more than two thirds of the surveyed children with 37.2% of the children concurrently infected with both *S. haematobium* and *S. mansoni*. Hookworm infections were also common. However, neither single infections with any investigated parasite species at any infection intensity, nor multiple species infections were associated with the maximal oxygen uptake VO_2_ max, which is a widely used parameter to determine and quantify physical fitness. VO_2_ max was only significantly related to children's age and sex and our cohort of children from Côte d'Ivoire presented with better physical fitness than children of the same age and sex in Canada.

Our data confirm previous studies showing that schistosomiasis and soil-transmitted helminthiasis are highly endemic in the Agboville area in south Côte d'Ivoire [38]–[41]. Interestingly, we found that children's physical activity in the current epidemiological setting of Côte d'Ivoire was, on average, considerably better than that of a large group of children from Canada. Indeed, the mean VO_2_ max, among our cohort of children was 52.7 ml kg^−1^ min^−1^, whereas a lower mean VO_2_ max (48.9 ml kg^−1^ min^−1^) had been observed in the aforementioned Canadian study [25]. Generally, school children in *Grand Moutcho* had a 3–7 ml kg^−1^ min^−1^ higher VO_2_ max than their age-matched Canadian counterparts (Table 1), which corresponds to a positive overall offset of about 8%, despite the fact that the Ivorian children were running during high ambient air temperatures (up to 43°C) on an unpaved schoolyard and some of them had only sandals or no shoes at all. Moreover, we could not find evidence that a helminth of *Plasmodium* infection, multiple species helminth infections, and heavy helminth infection intensities negatively impact on children's performance in a 20 m shuttle run test. Our findings are in contrast to two Kenyan studies published in the early 1990s [16], [17], but in line with other investigations carried out in the 1970s [11], [42], [43], and therefore raise the question as to why there is discrepancy between the widely held view that schistosomiasis and soil-transmitted helminthiasis prejudices physical fitness and the lack of consistent empirical data to support this claim.

The following points are offered for consideration. First, children in *Grand Moutcho* walk to school, day after day, often for several kilometers. Moreover, children are engaged in daily family chores, such as fetching water, help with subsistence agriculture and other physically demanding tasks. Compared to industrialized countries, where physical inactivity and other life-style modifiers have become important risk factors for ill-health [44]–[46], children living in rural parts of Africa still show high levels of physical activity. Second, what might also contribute to an increased fitness level of children in Africa is their potentially lower protein and fat intake compared to children in developed countries [47]. Hence, a limitation of our study is that neither nutritional parameters nor hemoglobin nor hematocrit levels of participating children were assessed. Third, according to Åstrand & Ryhming (1954) [48], neither sex nor anthropometric measures are significant predictors for physical fitness test results. In the present study, however, sex was associated with VO_2_ max with boys showing a statistically significantly higher mean value than girls. Moreover, there was a negative correlation between age and VO_2_ max capacity in the examined children. Fourth, children are at highest risk of helminth infections and, at the same time, easier to motivate for participation in a physical performance test than adults. This latter fact may also explain the relatively high voluntary compliance rate of 92.2% (i.e. 188 out of 204). Fifth, children attending school might not be fully representative for a specific epidemiological setting, as school-aged children from the poorest and furthest away households are less likely to be registered at school. These children might be at a higher risk of helminth and Plasmodium infections. Sixth, it is conceivable that those children suffering from a heavy helminth infection or clinical malaria rest at home because of abdominal pain, nausea or headache, and hence were absent at the time of the study. Seventh, an attrition analysis of the 32 children who had complete parasitological and clinical data but were excluded from the physical fitness test due to medical complaints (n = 26) or unreasonably low pulse rate (n = 6) revealed that they were not significantly different from the 156 included children in terms of sex, age, or parasite infection status. Nevertheless, 29 of the 32 children harbored at least one of the helminth species investigated, and hence it is possible that we excluded at least some individuals who suffered from severe disabilities attributable to their helmintic infections and thereby introduced a certain bias. Finally, our assumptions in the sample size calculation proofed to be too optimistic and mainly due to the unexpected high number of children, who had to be excluded from the physical fitness test because of medical complaints, the intended sample size could not be reached.

Currently, the results of the present study may support expert opinion that had assigned a minuscule disability weight for schistosomiasis, i.e., 0.005 for children aged 5–14 years and 0.006 for individuals aged 15 years and above on a scale from 0 (no disability) to 1 (death). These tiny disability weights, regardless of the schistosome species and infection intensity, are at the root of the low global burden estimate due to schistosomiasis, which, nonetheless, remains a heavily contested issue [6], [49]–[51]. Based on our findings one might indeed challenge the effect of a helminth infection on physical performance of school-aged children. Due to the lack of advanced chronic disease in this age group, low disability weights might be justified. However, schistosome infections that remain untreated ultimately lead to chronic morbid sequelae in later life, and hence the current emphasis on regular administration of anthelmintic drugs to entire at-risk populations is reasonable [52], [53].

Future efforts to further investigate the often subtle disabling effects of helminth infections are still needed, as the previous elaborations manifest. Hence, it would be interesting to investigate the dynamics of children's physical fitness in a pretest-posttest design with an intermittent treatment, or, even better, within the frame of continuous preventive chemotherapy campaigns, which was not feasible in the present study due to administrative and organizational constraints in the field. Such surveys should also apply quantitative tests instead of RDTs to diagnose *Plasmodium* species-specific infection intensities (i.e., thick and thin blood films for parasitemia appraisal) and consider nutritional status as well as hemoglobin values as potential co-factors influencing individual physical fitness. They could again use a similar maximal physical capacity test like the one used in this study and which had the advantage that a group of children could be processed at once without the requirement of any sophisticated equipment. However, to avoid the aforementioned problem that a large number of individuals suffering from the most severe attributable disabilities have to be excluded due to potentially harmful exertion, one could also think of low intensity physical fitness tests using, for example, pedo-, speedo- and/or accelerometers. In recent years, such high-quality measuring instruments have become available and handy, and they have been used extensively and reported as valid objective measures of physical activity [54]. The results of studies adhering to such further elaborated protocols could help to shed light on the true health consequences incurred by single and – hitherto even more neglected – multiple helminth species infections [55]. By assisting in the definition of appropriate disability weights for global burden of diseases calculations, such data would directly improve key stakeholders knowledge-base, and hence enable them to take well-informed decisions about future priority setting in the public health agenda.

## Supporting Information

Table S1 **Prevalence and intensities of helminth and** ***Plasmodium*** **spp. infections among 156 school children in Côte d'Ivoire.**
(DOC)
