# Supplementary material for: Effect of Schistosomiasis and Soil-Transmitted Helminth Infections on Physical Fitness of School Children in Côte d'Ivoire
Source: PLoS Negl Trop Dis. 2011 Jul 19;5(7):e1239. doi: 10.1371/journal.pntd.0001239 (PMC3139653; doi:10.1371/journal.pntd.0001239)
Supplement: Table S1 — Prevalence and intensities of helminth and Plasmodium spp. infections among 156 school children in Côte d'Ivoire. (DOC) [file pntd.0001239.s001.doc]

**Table S1. Prevalence and intensities of helminth and *Plasmodium* spp. infections among 156 school children in Côte d’Ivoire.**

|  |  |  | ***S. haematobium*^a^** | | | ***S. mansoni*^b^** | | | | **Hookworm^b^** | | | | ***A. lumbricoides*^b^** | | | | ***Plasmodium* spp.*^c^*** | |
| --- | --- | --- | --- | --- | --- | --- | --- | --- | --- | --- | --- | --- | --- | --- | --- | --- | --- | --- | --- |
| **Age (years)** | **Sex** | **n tested** | **n neg.** | **n light** | **n heavy** | **n neg.** | **n light** | **n mod.** | **n heavy** | **n neg.** | **n light** | **n mod.** | **n heavy** | **n neg.** | **n light** | **n mod.** | **n heavy** | **n neg.** | **n pos.** |
| 7 | M | 2 | 2 | 0 | 0 | 2 | 0 | 0 | 0 | 2 | 0 | 0 | 0 | 2 | 0 | 0 | 0 | 1 | 1 |
|  | F | 0 | 0 | 0 | 0 | 0 | 0 | 0 | 0 | 0 | 0 | 0 | 0 | 0 | 0 | 0 | 0 | 0 | 0 |
| 8 | M | 0 | 0 | 0 | 0 | 0 | 0 | 0 | 0 | 0 | 0 | 0 | 0 | 0 | 0 | 0 | 0 | 0 | 0 |
|  | F | 0 | 0 | 0 | 0 | 0 | 0 | 0 | 0 | 0 | 0 | 0 | 0 | 0 | 0 | 0 | 0 | 0 | 0 |
| 9 | M | 5 | 1 | 4 | 0 | 4 | 1 | 0 | 0 | 5 | 0 | 0 | 0 | 5 | 0 | 0 | 0 | 3 | 2 |
|  | F | 6 | 2 | 2 | 2 | 2 | 3 | 1 | 0 | 6 | 0 | 0 | 0 | 6 | 0 | 0 | 0 | 1 | 5 |
| 10 | M | 12 | 2 | 7 | 3 | 4 | 6 | 1 | 1 | 11 | 1 | 0 | 0 | 12 | 0 | 0 | 0 | 6 | 6 |
|  | F | 14 | 3 | 5 | 6 | 6 | 3 | 4 | 1 | 13 | 1 | 0 | 0 | 14 | 0 | 0 | 0 | 3 | 11 |
| 11 | M | 9 | 1 | 7 | 1 | 6 | 1 | 2 | 0 | 9 | 0 | 0 | 0 | 9 | 0 | 0 | 0 | 2 | 7 |
|  | F | 7 | 2 | 2 | 3 | 3 | 2 | 2 | 0 | 7 | 0 | 0 | 0 | 7 | 0 | 0 | 0 | 4 | 3 |
| 12 | M | 24 | 2 | 10 | 12 | 11 | 9 | 4 | 0 | 16 | 8 | 0 | 0 | 24 | 0 | 0 | 0 | 5 | 19 |
|  | F | 9 | 0 | 7 | 2 | 2 | 4 | 3 | 0 | 7 | 2 | 0 | 0 | 9 | 0 | 0 | 0 | 4 | 5 |
| 13 | M | 22 | 6 | 8 | 8 | 12 | 7 | 3 | 0 | 17 | 5 | 0 | 0 | 22 | 0 | 0 | 0 | 6 | 16 |
|  | F | 17 | 0 | 11 | 6 | 6 | 7 | 4 | 0 | 16 | 1 | 0 | 0 | 16 | 0 | 1 | 0 | 3 | 14 |
| 14 | M | 10 | 0 | 5 | 5 | 6 | 4 | 0 | 0 | 8 | 2 | 0 | 0 | 9 | 1 | 0 | 0 | 3 | 7 |
|  | F | 8 | 1 | 5 | 2 | 3 | 2 | 2 | 1 | 7 | 1 | 0 | 0 | 8 | 0 | 0 | 0 | 3 | 5 |
| 15 | M | 7 | 1 | 4 | 2 | 4 | 3 | 0 | 0 | 7 | 0 | 0 | 0 | 7 | 0 | 0 | 0 | 1 | 6 |
|  | F | 4 | 0 | 0 | 4 | 1 | 2 | 1 | 0 | 4 | 0 | 0 | 0 | 4 | 0 | 0 | 0 | 0 | 4 |
| 7-15 | M | 91 | 15 | 45 | 31 | 49 | 31 | 10 | 1 | 75 | 16 | 0 | 0 | 90 | 1 | 0 | 0 | 27 | 64 |
| 7-15 | F | 65 | 8 | 32 | 25 | 23 | 23 | 17 | 2 | 60 | 5 | 0 | 0 | 64 | 0 | 1 | 0 | 18 | 47 |
| 7-15 | Both | 156 | 23 | 77 | 56 | 72 | 54 | 27 | 3 | 135 | 21 | 0 | 0 | 154 | 1 | 1 | 0 | 45 | 111 |

Prevalence and intensities of helminth and *Plasmodium* spp. infections among 156 children attending grades 4-6 in the primary school of *Grand Moutcho II* and *III* near Agboville, south Côte d’Ivoire in early 2010. Results are stratified by age and sex and thresholds of helminth infection intensities are in accordance with WHO guidelines [37].

F, female; M, male.

^a^ Prevalence obtained by urine filtration method (2 consecutive urine samples per child).

^b^ Prevalence obtained by Kato-Katz method (2 consecutive stool samples per child with duplicate Kato-Katz thick smears per sample).

^c^ Prevalence obtained by rapid diagnostic test (RDT).
